# Prevalence dependence in model goodness measures with special emphasis on true skill statistics

**DOI:** 10.1002/ece3.2654

**Published:** 2017-01-12

**Authors:** Imelda Somodi, Nikolett Lepesi, Zoltán Botta‐Dukát

**Affiliations:** ^1^ MTA Centre for Ecological ResearchTihanyHungary; ^2^ Department of Plant Systematics, Ecology and Theoretical BiologyEötvös Loránd UniversityBudapestHungary; ^3^ National Adaptation CentreGeological and Geophysical Institute of HungaryBudapestHungary

**Keywords:** Cohen's kappa, model performance, predictive models, sample size, species distribution models

## Abstract

It has long been a concern that performance measures of species distribution models react to attributes of the modeled entity arising from the input data structure rather than to model performance. Thus, the study of Allouche et al. (*Journal of Applied Ecology*, 43, 1223, 2006) identifying the true skill statistics (TSS) as being independent of prevalence had a great impact. However, empirical experience questioned the validity of the statement. We searched for technical reasons behind these observations. We explored possible sources of prevalence dependence in TSS including sampling constraints and species characteristics, which influence the calculation of TSS. We also examined whether the widespread solution of using the maximum of TSS for comparison among species introduces a prevalence effect. We found that the design of Allouche et al. (*Journal of Applied Ecology*, 43, 1223, 2006) was flawed, but TSS is indeed independent of prevalence if model predictions are binary and under the strict set of assumptions methodological studies usually apply. However, if we take realistic sources of prevalence dependence, effects appear even in binary calculations. Furthermore, in the widespread approach of using maximum TSS for continuous predictions, the use of the maximum alone induces prevalence dependence for small, but realistic samples. Thus, prevalence differences need to be taken into account when model comparisons are carried out based on discrimination capacity. The sources we identified can serve as a checklist to safely control comparisons, so that true discrimination capacity is compared as opposed to artefacts arising from data structure, species characteristics, or the calculation of the comparison measure (here TSS).

## 1 Introduction

Measuring model performance (goodness) is a central issue in species distribution modeling (SDM, Guisan & Zimmermann, 2000) and predictive vegetation modeling (PVM, Franklin, 1995). There are three major tasks performance measures are used for: 1) comparing modeling techniques, typically using one dataset and the same species with each technique (e.g., Jones, Acker, & Halpern, 2010; Zurell et al., 2012), 2) comparing the performance of models of different species with one or more modeling techniques using one dataset (e.g., Coetzee, Robertson, Erasmus, Van Rensburg, & Thuiller, 2009; Engler et al., 2013; Pliscoff, Luebert, Hilger, & Guisan, 2014), and 3) when models of the same species are tested on different datasets (e.g., Randin et al., 2006; Ribeiro, Somodi, & Čarni, 2016).

In the first case, data properties are fixed and thus of less importance. Therefore, the actual prevalence in the data has no effect on the outcome of comparisons. On the contrary, when different species or prediction on different dataset is compared, characteristics of the data (including prevalence) may influence model performance.

Why is prevalence dependence a problem? If model goodness measures are used for tasks two and three, the intention is to compare how well the models reflect the species' environmental requirements (Elith & Graham, 2009; Robertson, Peter, Villet, & Ripley, 2003). Species with more distinct environmental requirements are expected to be modeled better (assuming that relevant predictors were included) compared to species with wide tolerance. If we want to assess the degree the models reflect true environmental requirements (as many has aimed at), we do not want rarity to interfere. For example, we have a model of a species and we test its discrimination capacity on test site A and test site B (task 3), and we expect to receive similar discrimination level. If the two sites differ in prevalence and a prevalence‐dependent measure is used, it will seem as if the model would have changed. It is similar when rating different species' models (task 2).

In fact, improving models of rare species, so that they reflect the environmental background better, has been a central issue lately (Lomba et al., 2010; Williams et al., 2009; Zimmermann, Edwards, Moisen, Frescino, & Blackard, 2007). We admit that there is a tendency that species with narrower tolerance are also less frequent, but it is not an absolute rule (Flather & Sieg, 2013; Kunin & Gaston, 1993). Besides autecological reasons, human activities may also account for a lower observed prevalence of a potentially common species.

Prevalence of different species may differ for two basic reasons: Either sampling points are fixed, but different species occur with different frequency, or presence information of species is independent because of a presence‐only collection scheme, which is often true for datasets originating from museum collections (Elith & Leathwick, 2007). It is difficult to imagine a project with real data, where each species has the same prevalence unless common species are resampled to low prevalence. The latter would however mean information reduction, which would be unnecessary if measures would not depend on prevalence.

Model goodness measures relate to calibration and discrimination ability (Lawson, Hodgson, Wilson, & Richards, 2014). While calibration measures the model's ability to match input data, discrimination reflects how well occurrences versus absences are found in independent data. Indices for discrimination ability include one truly threshold independent option (AUC, Hanley & McNeil, 1982) and several ones, where the basic idea is to find a threshold for the calculations of the index (kappa, true skill statistics [TSS], *F* score, Cohen, 1960; Allouche, Tsoar, and Kadmon (2006); Powers, 2011 respectively). The values of the index are then compared either at a threshold corresponding to the maximum or according to an equality criterion (e.g., TPR = TNR also called ROC‐based approach; Cantor, Sun, Tortolero‐Luna, Richards‐Kortum, & Follen, 1999). Although AUC is widely applied, many agree that it tends to be overoptimistic (Lobo, Jiménez‐Valverde, & Real, 2008; Shabani, Kumar, & Ahmadi, 2016), and therefore, it is often complemented by another measure of model goodness. This second measure used to be maximum kappa (Araújo & Luoto, 2007; Davidson, Hamilton, Boyer, Brown, & Ceballos, 2009; Guo & Liu, 2010). However, worries have been voiced about kappa being prevalence dependent and thus potentially providing misleading information (McPherson, Jetz, & Rogers, 2004; Pontius & Millones, 2011). Lately, TSS has been applied instead (also in prestigious packages as BIOMOD, Thuiller, Lafourcade, Engler, & Araújo, 2009) as Allouche et al. (2006) claimed that it is insensitive to prevalence differences. Nonetheless, reaction of TSS has been observed in relation to prevalence differences in actual studies (Allouche et al., 2006; Hanspach, Kühn, Pompe, & Klotz, 2010). Some other threshold dependent measures (*F* score, Drake, Randin, & Guisan, 2006; Powers, 2011) are also available, but their use is much more restricted then that of TSS. Please note that TSS exists under a wide variety of synonyms, typically used outside ecology (see also Wilks, 2011) except for the last one mentioned: Youden index (Youden, 1950), Peirce Skill Score (Peirce, 1884), Kuipers Skill Score (Murphy & Daan, 1985), Sum of Sensitivity and Specificity (SSS, Liu, White, & Newell, 2013). It is also noteworthy that TSS is most often applied in the form of maximum TSS over all possible probability cutoffs (e.g., in the BIOMOD package) and advocated in reviews in this form (Liu, Berry, Dawson, & Pearson, 2005; Liu et al. 2016).

Motivated by the observed prevalence effects in TSS, we aimed at finding reasons, why such pattern may arise. We specifically set the following aims to:

- revisit Allouche et al. (2006) if their arguments (whether theoretical or simulation‐based) appropriately prove that TSS is independent of prevalence
- explore possible manifestations of prevalence dependence in theory
- determine whether and how TSS is prevalence dependent
- search for the source of prevalence dependence of TSS experienced in practice.

## 2 Theoretical considerations

### 2.1 A critique to the design of Allouche et al. (2006)

The true skill statistics is defined based on the components of the standard confusion matrix representing matches and mismatches between observations and predictions (Fielding & Bell, 1997; Table 1.).

**Table 1:** Confusion matrix of matches and mismatches of predictions and observations

Observation	Prediction		
	1	0	Σ
1	True positives (TP)	False negatives (FN)	No. positive observations (*P* = π*N)
0	False positives (FP)	True negatives (TN)	*N* − *P*
Σ	Number of positive predictions (*S*)	*N* − *S*	Total number of observations (*N*)

True skill statistics is defined as

TSS=TPR+TNR−1,

Where

$$\mathrm{TPR}=\frac{\mathrm{TP}}{\mathrm{TP}+\mathrm{FN}}$$

$$\mathrm{TNR}=\frac{\mathrm{TN}}{\mathrm{TN}+\mathrm{FP}}$$

The literature refers to TPR as true‐positive rate or sensitivity, while to TNR as true‐negative rate or specificity (Fielding & Bell, 1997). In the rare case, when predictions are binary, computation of the confusion matrix is straightforward. If there are probabilistic predictions, the goodness measure relying on the contingency table is calculated by converting the probabilities into presence and absence predictions. This is usually carried out by carrying out such a conversion at evenly spaced values of the probability spectrum (e.g., 0.1, 0.2, …, 0.9). These values are termed cutoffs or thresholds.

Allouche et al. (2006) claim to have randomized their models; however, they only randomized their confusion matrix. They held the value of TPR and TNR constant. If TPR and TNR, or their sum, is held constant, TSS cannot vary theoretically.

Allouche et al. (2006) set: TPR = TNR = 0.8 or TPR = 0.7 and TNR = 0.9 or the opposite way. Thus

TSS = TPR + TNR − 1 = 0.8 + 0.8 − 1 = 0.6

or TSS = TPR + TNR − 1 = 0.7 + 0.9 − 1 = 0.6

Therefore, whatever the prevalence, the result is 0.6, as it is also clear from Figure 1 in Allouche et al. (2006). The low‐level variation in the TSS value in the figure is due to the constraint that numbers in the cells of the contingency table (including true‐positive and true‐negative cases) have to be integer; thus, actual TPR/TNR may slightly differ from the theoretical values.

**Figure 1:**
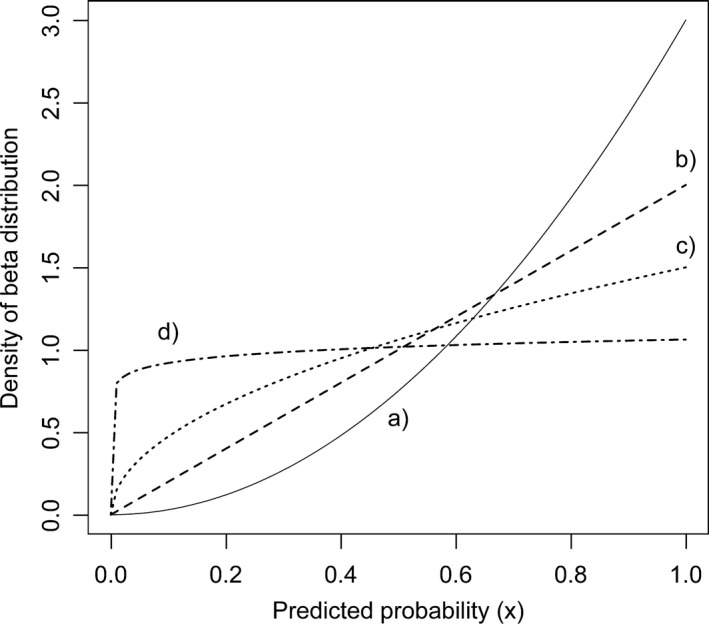
Subcases of beta distribution with parameters defined in Table 6. The sampling of probability values for presence “observations” is carried out according to these curves in our simulations. The individual predicted probability values appear in our simulated predictions with such densities. Lines represent: a) quadratic, b) linear, c) square root, d) 1/16th power curve

### 2.2 Redefinition of prevalence dependence

As Allouche et al. (2006) did not appropriately prove that TSS is independent of prevalence and empirical experience indicates such an effect, there is a need to revisit prevalence dependence in TSS. The usual interpretation of prevalence dependence in distribution modeling (Lawson et al., 2014; Manel, Williams, & Ormerod, 2001; McPherson et al., 2004; Santika, 2011) is that the value of the index should be constant over prevalence ranges if model goodness is constant. We follow this definition, but it should be mentioned that alternative definitions of prevalence dependence could be developed. For example, an index could be regarded as prevalence independent, if its range (i.e., maximum and minimum values) does not depend on prevalence (cf. independence of beta‐diversity from alpha‐ and gamma‐diversity; Jost, 2007).

The problem is how to measure model goodness exclusively without the confounding effects arising from data structure and especially prevalence differences. Lawson et al. (2014) pointed out that there is a distinction whether a performance measure quantifies model calibration or discrimination. In line with their opinion and taking into account that TSS measures discrimination capacity, we are targeting this model feature in our considerations. Thus, we consider two models equally good if they are characterized by same rate of discrimination errors (error rates of FP and FN). We examine two types of influences on TSS: the discrimination capacity of the model (1 − *e*) and prevalence (π = *P*/*N*) in the data. In all our calculations, we fixed the total sample size (*N*); therefore, the ratio of presence observations and total number of observations (prevalence, π = *P*/*N*) only depends on the absolute number of presence observations (*P* = N*π). Therefore, if *P* is present in any equation leading to TSS, it also indicates prevalence dependence.

While the representation of *e* in the equations is thus desirable (TSS was designed to reflect that), if *P* or π is in the equation, then prevalence also matters and can confound discrimination effectiveness.

The majority of the currently available model goodness measures and especially Kappa and TSS rely on a dichotomic representation of site occupancy. Therefore, they actually reduce the problem to a dichotomic representation of habitat suitability: Each of the locations is either suitable or unsuitable for the organism. The fact that we have no actual information on this suitability has not been taken into account yet, even though many of the predictive models are targeting the mapping of this suitability. Nonetheless, all estimations have errors, which can arise if 1) the model does not precisely predict suitability (for example, because not all relevant variables were measured). This kind of error is the most commonly considered error type (Guisan & Zimmermann, 2000; Pearce & Ferrier 2000). Discrimination capacity measures are expected to reflect the degree of this error and this error only. However, as Hirzel and Le Lay (2008) have introduced, there is another possible source of error: 2) the observed pattern does not fully reflect the suitability pattern, for example, due to sink populations or other components of metapopulation dynamics. We assume that the two kinds of errors do not extinguish each other (or would do so under very specific conditions only); therefore, we examine their cases separately.

### 2.3 Binary considerations

Although less frequent in practice, we first examine the case when not only observations, but also predictions are binary. If the model goodness measure appears independent of prevalence in such a case, the second step is the examination whether any prevalence dependence appears if continuous predictions are considered.

We take the strategy of proceeding from simple cases toward complex ones. We assume that if prevalence dependence appears in a simple case, it is unlikely that it disappears in the corresponding more complex cases.

In case 1), we assume that the observed pattern coincides with the suitability. In such a case, the contingency table takes the form presented in Table 2.

$$\mathrm{TPR}=\frac{\mathrm{TP}}{\mathrm{TP}+\mathrm{FN}}=1-e_{1}$$

$$\mathrm{TNR}=\frac{\mathrm{TN}}{\mathrm{TN}+\mathrm{FP}}=1-e_{2}$$

$$\mathrm{TSS}=\mathrm{TPR}+\mathrm{TNR}-1=1-e_{1}-e_{2}$$

**Table 2:** Confusion matrix of matches and mismatches of predictions and observations assuming different rates of false‐negative and false‐positive errors, *e*_1_ and *e*_2_

Observation	Prediction		
	1	0	Σ
1	TP = (1 − *e*_1_) *P*	FN = *e*_1_ *P*	*P* = π*N*
0	FP = *e*_2_(*N* − *P*)	TN = (1 − *e*_2_) (*N* − *P*)	*N* − *P*
Σ	*S*	*N* − *S*	*N*

Applying our definition of model goodness (i.e., the opposite of the level of error rates) to these equations, TSS is prevalence independent, as its value can be calculated from the two error rates (*e*_1_ and *e*_2_) without using the prevalence value. This form of prevalence dependence is the usually considered and tested from Manel et al. (2001), McPherson et al. (2004), Santika (2011), Lawson et al. (2014). Prevalence dependence of kappa has been proved for this case with equal error rates (i.e., *e*_1_ = *e*_2_) by McPherson et al. (2004).

Let us examine case 2 now, when we disregard potential weaknesses of the models but allow misleading observations, that is, allow the observed distribution pattern to be different from the habitat suitability pattern. Such situation can arise, for example, from intense metapopulation dynamics, sink subpopulations, or a transient animal being difficult to spot in the habitat. Differences between the suitability and observations can appear as a) missed presences, b) fallacious presences, and c) fallacious absences (Hirzel & Le Lay, 2008). The first two contribute to false‐positive predictions, while the last one appears as false negative, although this may be mitigated by missed presences.

- Firstly, we examine the case when there are missed presences only; that is, some of the presences are not detected even though the place is suitable and the species lives there. In the simplest case, the sampling error (i.e., the rate of missed presences denoted by *e*; Table 3) is constant, and thus, this error itself is independent from prevalence. (We do not miss more presences if the species is rare.)

**Table 3:** Contingency table when the model is assumed to be perfect, but there are missed presences in the observations. “*e*” denotes the rate of missed presences

Observation	Prediction		
	1	0	Σ
1	TP = (1 − *e*) *S*	FN = 0	*P* = π*N*
0	FP = *eS*	TN = (*N* − *S*)	*N* − *P*
Σ	*S* = *P*/(1 − *e*)	*N* − *S*	*N*

$$\mathrm{TPR}=\frac{\mathrm{TP}}{\mathrm{TP}+\mathrm{FN}}=1$$

$$\mathrm{TNR}=\frac{\mathrm{TN}}{\mathrm{TN}+\mathrm{FP}}=\frac{N-S}{N-P}=\frac{N-P/\left(1-e\right)}{N-P}=\frac{\left(1-e-\pi \right)\left(1-\pi \right)}{1-e}$$

$$\mathrm{TSS}=\mathrm{TPR}+\mathrm{TNR}-1=\frac{N-P/\left(1-e\right)}{N-P}=\frac{\left(1-e-\pi \right)\left(1-\pi \right)}{1-e}$$

Even if the level of error does not depend on prevalence directly, TSS does appear to depend on prevalence (π) according to the equations above. Therefore, TSS differences may arise for species with different π even though we fixed the rate of missed presences (constant *e*) and did not allow any other error source.

Secondly, let us consider fallacious absences (i.e., the species is not present even though the habitat is suitable) as the only source of error. As in metapopulation dynamics, we can assume that the number of false‐positive cases is proportional to the number of suitable sites (i.e., the error rate is constant; Table 4.). From a mathematical point of view, this case is equivalent to the previous one.

**Table 4:** Contingency table when the model is assumed to be perfect, but there are fallacious absences in the observations

Observation	Prediction		
	1	0	Σ
1	TP = *P*	FN = 0	*P*
0	FP = *eS*	TN = (*N* − *S*)	*N* − *P*
Σ	*S* = *P*/(1 − *e*)	*N* − *S*	*N*
Thirdly, let us examine when fallacious presences are present and there is no other source of error. There are two reasonable alternative assumptions regarding error rates:

i. Some proportion of presences is a fallacious presence. This is equivalent to case 1, if *e*_2_ = 0. We have proven that TSS is prevalence independent in this case. (ii) The number of fallacious presences is proportional to the number of unsuitable sites (Table 5). In this case, TSS is prevalence dependent:

**Table 5:** Contingency table when the model is assumed to be perfect, but there are fallacious presences and their amount is proportional to the number of unsuitable sites in the observations

Observation	Prediction		
	1	0	Σ
1	TP = *P* − *e* (*N* − *S*) = *S*	FN = *e* (*N* − *S*)	*P*
0	FP = 0	TN = *N* − *P*	*N* − *P*
Σ	*S*	*N* − *S*	*N*

From Table 5, it follows that

S=(P−eN)/(1+e),

Thus,

$$\mathrm{TPR}=\frac{\mathrm{TP}}{\mathrm{TP}+\mathrm{FN}}=\frac{S}{P}=\frac{P-eN}{P\left(1+e\right)}=\frac{\pi -e}{\pi \left(1+e\right)}$$

$$\mathrm{TNR}=\frac{\mathrm{TN}}{\mathrm{TN}+\mathrm{FP}}=1$$

$$\mathrm{TSS}=\mathrm{TPR}+\mathrm{TNR}-1=\frac{\pi -e}{\pi \left(1+e\right)}$$

Our findings regarding the prevalence dependence of TSS is summarized in Table 6.

**Table 6:** Is there prevalence dependence in TSS? Answers for cases examined in our study

Species occupy suitable sites only, and model goodness changes.		Species occupy unsuitable sites also, and model goodness is fixed (for our analysis). Binary predictions considered only. Source of species' distribution difference:		
Binary predictions	Continuous predictions	Missed presence	Fallacious absence	Fallacious presence
No	Yes for small sample size, No for large sample size	Yes	Yes	Yes, except if the rate of fallacious presences is proportional to the number of unsuitable sites

### 2.4 The case of continuous predictions

Having explored prevalence dependence of binary predictions, we examine whether binarization has any influence on prevalence dependence. First of all, there is no need to examine cases, where there has been prevalence dependence discovered in the binary case, as continuous predictions are reduced to binary cases at regular cutoffs to provide a distribution of goodness values, from which usually the maximum is chosen. If there is already prevalence dependence in the binary case, it is unlikely that repeated application of the same principle would eliminate the effect. It was case 1, the most popular interpretation of prevalence dependence in fact (when the species is assumed to occupy suitable sites only), which showed no prevalence dependence. However, as detailed in the Introduction, empirical prevalence dependence has been observed. Therefore, we examine whether binarization induces such an effect.

We can formulate TPR and TNR as conditional probabilities given a binary prediction as already pointed out by Lawson et al. (2014):

TPR=P(x=1|speciespresent)

TNR=P(x=0|speciesabsent)

where *x* denotes the predicted value.

If we have continuous probabilities as prediction, the equations are as follows:

$$\mathrm{TPR}=P\left(x>x_{c}|\mathrm{species}\;\mathrm{present}\right)$$

$$\mathrm{TNR}=P\left(x\leq x_{c}|\mathrm{species}\;\mathrm{absent}\right)$$

where *x*_c_ refers to the cutoff value corresponding to maximum TSS.

Let *F*_1_ and *F*_0_ denote the conditional distribution functions of predicted values conditional on the presence and absence of the species, subscripts refer to presence (1) and absence (0), respectively. The expected value of TPR, TNR, and TSS is as follows:

$$\begin{array}{cc} E\;(\mathrm{TPR}) & =P\left(x>x_{c}|\mathrm{species}\;\mathrm{present}\right)=1-P\left(x\leq x_{c}|\mathrm{species}\;\mathrm{present}\right)\\ & =1-F_{1}\left(x_{c}\right) \end{array}$$

$$\begin{array}{cc} E\;(\mathrm{TPR}) & =P\left(x>x_{c}|\mathrm{species}\;\mathrm{present}\right)=1-P\left(x\leq x_{c}|\mathrm{species}\;\mathrm{present}\right)\\ & =1-F_{1}\left(x_{c}\right) \end{array}$$

$$E\;\left(\mathrm{TNR}\right)=P\left(x\leq x_{c}|\mathrm{species}\;\mathrm{absent}\right)=F_{0}\left(x_{c}\right)$$

$$E\;\left(\mathrm{TSS}\right)=F_{0}\left(x_{c}\right)-F_{1}\left(x_{c}\right)$$

As usually the cutoff corresponding to the maximum value of TSS is used, we inspect the prevalence dependence of this measure. The maximum of the expected value of TSS is where the derivative is 0.

$$\frac{\partial E\left(\mathrm{TSS}\right)}{\partial x_{c}}=\frac{\partial F_{0}\left(x_{c}\right)}{\partial x_{c}}-\frac{\partial F_{1}\left(x_{c}\right)}{\partial x_{c}}=0$$

The derivatives of *F*_0_ and *F*_1,_ that is, the density functions, will be referred to as *f*_0_ and *f*_1_; thus, TSS is maximal where

$$f_{0}\left(x_{c}\right)-f_{1}\left(x_{c}\right)=0$$

If the theoretical curves were known and the cutoff was based on them or any other a priori threshold setting method was chosen, TSS would indeed be prevalence independent. In practice, however, the cutoff is determined from the data. Due to this, the mean of TSS maxima will be higher than the expected value, because we only choose a maximum value other than the one corresponding to the theoretical cutoff if the former is higher. Thus, the mean of TSS maxima is a biased estimate of the theoretical TSS. The bias is due to the cumulative frequency distribution being different from the theoretical distribution function. We have two theoretical distribution functions with two corresponding cumulative frequency functions. The theoretical distribution function and the cumulative frequency function increasingly resemble each other with increasing sample size. If the sample size is fixed, but prevalence changes, the fit of the cumulative distribution function to the theoretical distribution function improves for one of the conditional distribution, but deteriorates for the other. If the improvement/deterioration depends on prevalence in a nonlinear manner, they do not extinguish the effect of each other, which may result in the prevalence dependence observed. We tested this effect with numerical simulations.

## 3 Simulation methods and results

### 3.1 Methods

We constructed model scenarios where two aspects varied, discrimination capacity and prevalence. We varied prevalence as the proportion of presences in the observations from 0.05 to 0.95 in increments of 0.05. This corresponds to the approach of Allouche et al. (2006) and other papers studying the effect of prevalence on kappa (Manel et al., 2001; McPherson et al., 2004). To observe the effect of sample size, the following sizes were applied: 100, 1,000, 10,000. Presence or absence was allocated to this amount of observations so as to produce the prevalence desired.

Predicted probability values were randomly chosen from the beta distribution with parameters given in the Table 7 representing different model goodness scenarios. Density functions of predicted probabilities for presences (*f*_1_) and absences (*f*_0_) were defined by the following general formula:

$$f\left(x\right)=\left\{\begin{array}{cc} \frac{{x^{\alpha -1}\left(1-x\right)}^{\beta -1}}{\int_{0}^{1}t^{\alpha -1}{\left(1-t\right)}^{\beta -1}dt}, & \mathrm{if}\;x\in [0,1]\\ 0, & \mathrm{otherwise} \end{array}\right.$$

where *x* corresponds to possible values of the suitability estimate, while α and β are the parameters of the distribution. Parameters has been chosen so that *f*_0_(*x*) = 1 − *f*_1_(*x*) if $x\in \left[0,1\right]$, and it is always true that higher predicted probabilities are chosen more frequently than lower ones for presences, while there is an opposite trend for absences. We will refer to the models according to the function in the nominator of *f*_1_(*x*): a) quadratic, b) linear, c) square root, d) a power of 1/16 (Figure 1) curves. The steepness of function in the nominator of *f*_1_(*x*) represents the discrimination power. Steepness patterns were selected so as to present contrasting distribution of predictions and thus to represent different discrimination powers. The quadratic curve corresponds to the best model, where low probabilities are disproportionately more often assigned to absences, while high probabilities to presences. The linear curve represents medium model performance, while the application of the square root function results in a weakly discriminating model, where medium probabilities are assigned both to presences and absences in most cases. The 1/16th power corresponds to extreme weak discrimination. TSS was calculated at 19 cutoffs (thresholds) equally spaced along the probability spectrum (0.05–0.95) for each prevalence ratio and model goodness scenarios. This was repeated 1,000 times for each combination to assess variation. The mean of the maximum TSS values was calculated for each combination of model goodness scenarios and prevalence values. Calculations were carried out in the R Statistical Environment (R Core Team 2014).

**Table 7:** The *f*_0_ and *f*_1_ functions used in our simulations are specific cases of the beta distribution if α = 1 or β = 1. The table shows the corresponding other parameter of the beta distribution producing the probability function of selecting a certain probability value for presence observations. Selections for absence observations follow the opposite trend. The rbeta function in R generates random numbers with such distributions (Appendix S1)

Curve type	*f*_1_	*f*_0_
Quadratic	α = 3, β = 1	β = 3, α = 1
Linear	α = 2, β = 1	β = 2, α = 1
Square root	α = 1.5, β = 1	β = 1.5, α = 1
16^th^ root	α = 17/16, β = 1	β = 17/16, α = 1

### 3.2 Results

**Figure 2:**
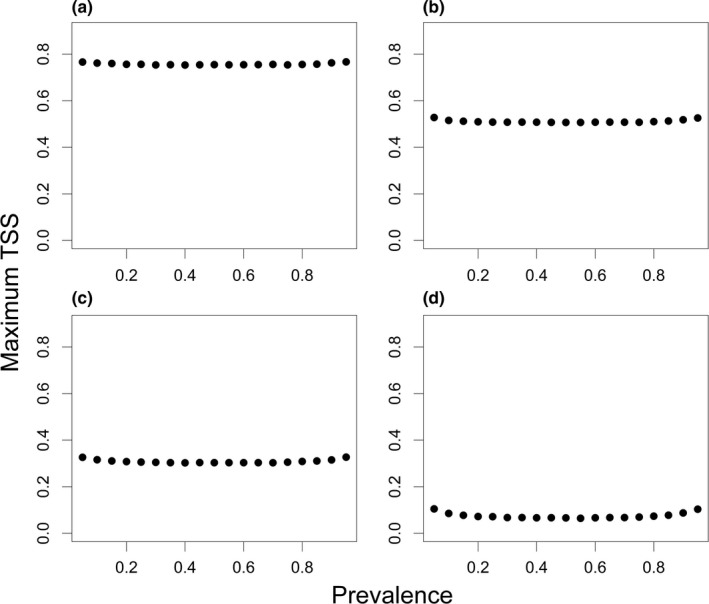
Demonstration of the dependence of the maximum value of TSS on prevalence. The ratio of presences and absences in the observations (prevalence) was varied from 0.05 to 0.95 in increments of 0.05. Average maximum values from 1,000 simulations are shown for four model scenarios (a)–(d). For details, see Fig 1

**Figure 3:**
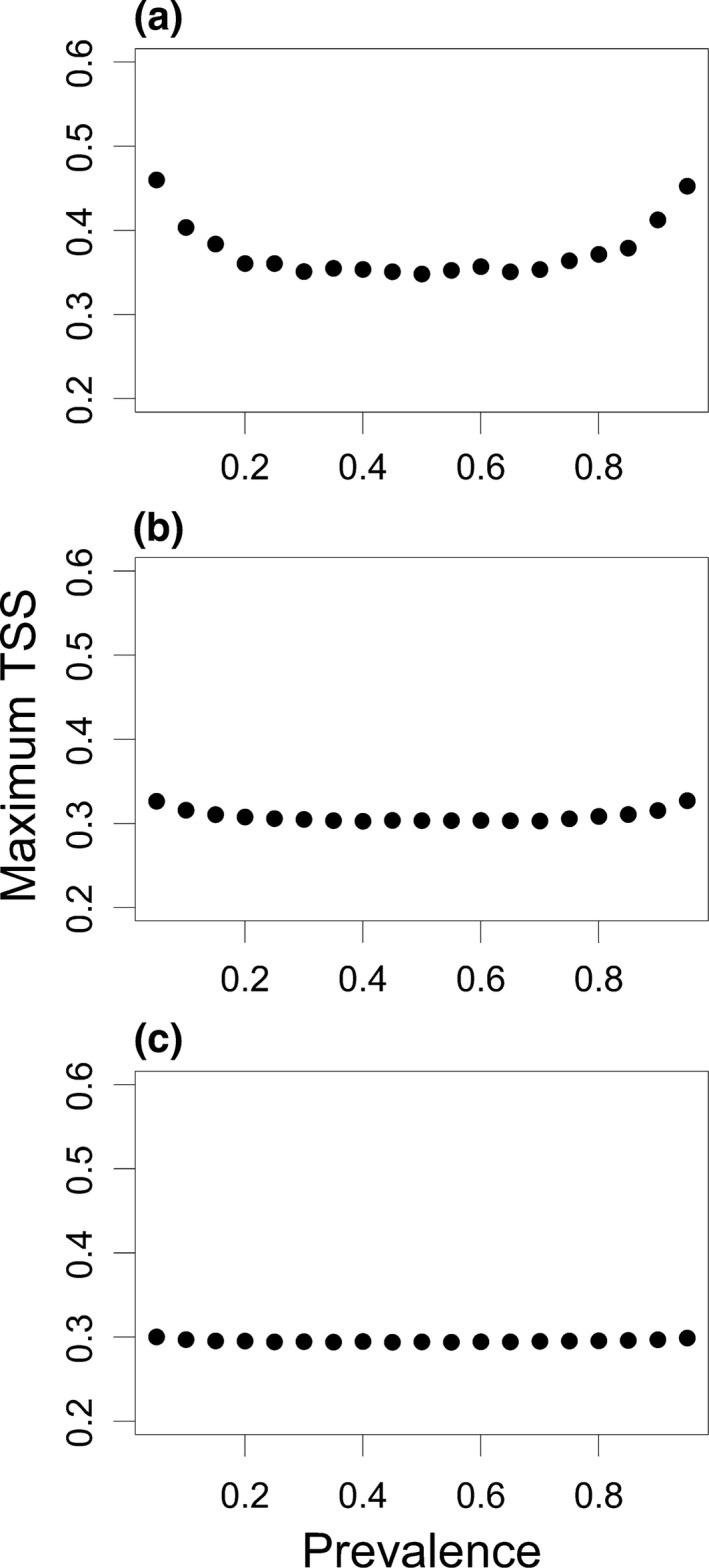
Decrease of prevalence effect on maximum TSS with increasing sample size. Sample size equals to (a) 100, (b) 1,000, (c) 10,000. The pattern is similar for all model goodness scenarios; here, sampling according to the square root function (medium model quality) is used as an example

We found a response to prevalence changes in the maximum value of TSS for small sample sizes (Figures 2 and 3), which however decreased with an increase in sample size and approached the theoretically expected value. Sample size of 10,000 eliminated any TSS bias even for the worst model even with lowest prevalence corresponding 500 presences. Sample size of 1,000 with 50 presences showed prevalence dependence for the worst model only; thus, already this sample size can be applied with confidence for reasonably performing models.

The dependence at low sample size had an U‐shaped form, implying that the same model goodness can result in higher maximum TSS solely due to a low or high prevalence if sample size is low (corresponding to a rare or common species; Figure 2.). The dependence on prevalence increased with decreasing model quality at constant sample sizes.

## 4 Discussion

We found that prevalence dependence is absent in TSS only under strict assumptions and large sample sizes. This is in contrast with actual use of TSS, when these assumptions are often violated. Allouche et al. (2006) used a flawed design; therefore, their results are not relevant. However, using their assumptions, TSS is indeed not prevalence dependent. Nonetheless, there is a tendency for prevalence dependence observations in TSS (Allouche et al., 2006; Lawson et al., 2014; McPherson et al., 2004).

Causes of prevalence dependence could be retraced in our study either to 1) a lack of ideal association of species with suitable sites or 2) the use of the maximum value of TSS for cutoff selection and especially at small sample sizes.

1. Previous considerations of prevalence dependence in general assumed that species occupy all suitable sites and suitable sites only. This is often not the case (Hirzel & Le Lay, 2008). This narrow assumption had no significance regarding prevalence dependence of the previously more common kappa, as it proved to be prevalence dependent even under those idealistic assumptions (McPherson et al., 2004). If species behavior does not follow that assumption, the prevalence dependence is not likely to diminish. However, we found that in the binary case (which is also equivalent to a predetermined cutoff), TSS is indeed not prevalence dependent (although not for the reason Allouche et al., 2006 gave). Nonetheless, this only holds if a species closely follows the suitability pattern. Ideally, we want to evaluate the capacity of a model to trace suitability pattern and when we compare species want to compare this property. However, we found that if species are differently detectable (differ in the proportion of missed presences) or tend to leave suitable space open (fallacious absences) or tend to occur at unsuitable places (fallacious presences) to a degree differing, these features might mix up with model discrimination capacity and may lead to artefacts in comparisons.

There is abundant evidence against species closely following suitability patterns, including metapopulation theory (Hanski, 1991), extinction debt (Tilman, May, Lehman, & Nowak, 1994), and other considerations (Gu & Swihart, 2004). Such mechanisms may be behind “species characteristics” influencing model performance such as in Hernandez, Graham, Master, and Albert (2006) and Hanspach et al. (2010) and may also account for the prevalence dependence seen in Allouche et al. (2006)'s Figure 2.

We offer no solution yet; our aim here is to draw the attention that these aspects need to be considered when making comparisons. The tendency for the appearance of missed presences is related to the life strategy of the species, so it might not be a problem if models of similar species are compared (e.g., several species of trees: Zimmermann et al., 2009), but comparison between species with great differences (butterflies vs. plants; e.g., Hanspach et al., 2014) may become significant.

Tendency for fallacious absences and presences is likely in connection with the degree of involvement of metapopulation dynamics in the species' distribution. Fallacious absences reflect a population structure, where empty suitable patches are a constant proportion in the landscape (cf. Levin's model, Pásztor, Botta‐Dukát, Magyar, Meszena, & Czárán, 2016; Husband & Barrett, 1998), while fallacious presences can reflect sink populations (e.g., Ficetola, Thuiller, & Padoa‐Schioppa, 2009).

The sample size effect has been observed in relation to the use of maximum TSS, which is most widespread in the literature in relation to TSS use (a few recent examples: Zurell et al., 2012; Gallardo & Albridge 2013, Baross et al. 2015). It is also one of the default measures in BIOMOD (Thuiller et al., 2009), one of the most widespread SDM tool and also propagated in reviews (Liu 2005; Liu, Newell, & White, 2016). While users of max TSS still assume that they use a prevalence independent measure, we observed as large differences as almost 0.2 in the average maximum TSS due to differences in prevalence only even in “good models” at the lowest sample size. Differences in maximum TSS as small as 0.001 and 0.06 have been interpreted as the model with the higher TSS being superior to the one with the lower maximum value (Coetzee et al., 2009 and Zurell et al., 2012, respectively). Therefore, the level of influence of prevalence detected for low sample sizes has a message for the practice, too.

One could argue that lower sample sizes used in our simulations (100 observations with 5–95 presences within) are extreme, but several similar examples can be found (e.g., Hernandez et al., 2006; Papeş & Gaubert, 2007; Williams et al., 2009; Wisz et al., 2008). Species' distribution models of rare plants are frequent target of research (Engler, Guisan, & Rechsteiner 2004; Guisan et al., 2006; Zimmermann et al., 2007; Williams et al., 2009), where both extreme prevalence and sample sizes occur. According to our results, in such cases, the effect of data structure may be particularly severe, and therefore, automatically applying maximum TSS for across‐species or across‐sites comparison may lead to erroneous conclusions. We agree with Lobo et al. (2008) that in such cases, indices should be adjusted to the case studied taking into account the potential effect of prevalence on the indices.

It is also worth to note that prevalence dependence does not affect the comparison of different models of a single species from a single dataset. Thus, our finding does not affect model type comparisons for one species with one dataset, such as the ensemble modeling approach in BIOMOD, which heavily relies on TSS (Thuiller et al., 2009).

## 5 Conclusions

The redefinition of prevalence dependence has brought a wider range of interpretations and explanations to attention. Sources of prevalence dependence have to be considered when evaluating models of different objects (while it is no concern when different models of the same object with the same data points are compared). We found three sources of prevalence dependence not yet considered, arising for an incomplete reflection of habitat suitability in species' distribution: different degree of missed presences, fallacious absences, and fallacious presences per species. Another source of potential prevalence dependence was the use of maximum value over the predicted probability continuum for comparisons (maximum TSS). We found three risk factors for prevalence dependence even when assuming species perfectly mirroring suitability but using maximum TSS for across‐species comparisons: rare or very common species, small sample sizes, and weak models.

## Conflict of Interest

None declared.

## References

[ece32654-bib-0001] Allouche, O. , Tsoar, A. , & Kadmon, R. (2006). Assessing the accuracy of species distribution models: Prevalence, kappa and the true skill statistic (TSS). Journal of Applied Ecology, 43, 1223–1232.

[ece32654-bib-0002] Araújo, M. B. , & Luoto, M. (2007). The importance of biotic interactions for modelling species distributions under climate change. Global Ecology and Biogeography, 16, 743–753.

[ece32654-bib-0058] Barros, M. J. , Silva‐Arias, G. A. , Fregonezi, J. N. , Turchetto‐Zolet, A. C. , Iganci, J. R. , Diniz‐Filho, J. A. F. , & Freitas, L. B. (2015). Environmental drivers of diversity in Subtropical Highland Grasslands. Perspectives in Plant Ecology, Evolution and Systematics, 17(5), 360–368.

[ece32654-bib-0003] Cantor, S. B. , Sun, C. C. , Tortolero‐Luna, G. , Richards‐Kortum, R. , & Follen, M. (1999). A comparison of C/B ratios from studies using receiver operating characteristic curve analysis. Journal of Clinical Epidemiology, 52(9), 885–892. 10529029 10.1016/s0895-4356(99)00075-x

[ece32654-bib-0004] Coetzee, B. W. , Robertson, M. P. , Erasmus, B. F. , Van Rensburg, B. J. , & Thuiller, W. (2009). Ensemble models predict Important Bird Areas in southern Africa will become less effective for conserving endemic birds under climate change. Global Ecology and Biogeography, 18, 701–710.

[ece32654-bib-0005] Cohen, J. (1960). A coefficient of agreement for nominal scales. Educational and Psychological Measurement, 20, 37–46.

[ece32654-bib-0006] Davidson, A. D. , Hamilton, M. J. , Boyer, A. G. , Brown, J. H. , & Ceballos, G. (2009). Multiple ecological pathways to extinction in mammals. Proceedings of the National Academy of Sciences, 106, 10702–10705. 10.1073/pnas.0901956106 PMC2705575 19528635

[ece32654-bib-0007] Drake, J. M. , Randin, C. , & Guisan, A. (2006). Modelling ecological niches with support vector machines. Journal of Applied Ecology, 43(3), 424–432.

[ece32654-bib-0008] Elith, J. , & Graham, C. H. (2009). Do they? How do they? WHY do they differ? On finding reasons for differing performances of species distribution models. Ecography, 32(1), 66–77.

[ece32654-bib-0009] Elith, J. , & Leathwick, J. (2007). Predicting species distributions from museum and herbarium records using multiresponse models fitted with multivariate adaptive regression splines. Diversity and Distributions, 13(3), 265–275.

[ece32654-bib-0059] Engler, R. , Guisan, A. , & Rechsteiner, L. (2004). An improved approach for predicting the distribution of rare and endangered species from occurrence and pseudo‐absence data. Journal of Applied Ecology, 41(2), 263–274.

[ece32654-bib-0010] Engler, R. , Waser, L. T. , Zimmermann, N. E. , Schaub, M. , Berdos, S. , Ginzler, C. , & Psomas, A. (2013). Combining ensemble modeling and remote sensing for mapping individual tree species at high spatial resolution. Forest Ecology and Management, 310, 64–73.

[ece32654-bib-0011] Ficetola, G. F. , Thuiller, W. , & Padoa‐Schioppa, E. (2009). From introduction to the establishment of alien species: Bioclimatic differences between presence and reproduction localities in the slider turtle. Diversity and Distributions, 15, 108–116.

[ece32654-bib-0012] Fielding, A. H. , & Bell, J. F. (1997). A review of methods for the assessment of prediction errors in conservation presence/absence models. Environmental Conservation, 24, 38–49.

[ece32654-bib-0013] Flather, C. H. , & Sieg, C. H. (2013). Species rarity: Definition, causes and classification In Raphael M. G., & Molina R. (Eds.), Conservation of rare or little‐known species: Biological, social, and economic considerations (pp. 40–66). Washington: Island Press.

[ece32654-bib-0014] Franklin, J. (1995). Predictive vegetation mapping: Geographic modeling of biospatial patterns in relation to environmental gradients. Progress in Physical Geography, 19, 474–499.

[ece32654-bib-0062] Gallardo, B. , & Aldridge, D. C. (2013). Evaluating the combined threat of climate change and biological invasions on endangered species. Biological Conservation, 160, 225–233.

[ece32654-bib-0015] Gu, W. , & Swihart, R. K. (2004). Absent or undetected? Effects of non‐detection of species occurrence on wildlife–habitat models. Biological Conservation, 116, 195–203.

[ece32654-bib-0016] Guisan, A. , Broennimann, O. , Engler, R. , Vust, M. , Yoccoz, N. G. , Lehmann, A. , & Zimmermann, N. E. (2006). Using niche‐based models to improve the sampling of rare species. Conservation Biology, 20, 501–511. 16903111 10.1111/j.1523-1739.2006.00354.x

[ece32654-bib-0017] Guisan, A. , & Zimmermann, N. E. (2000). Predictive habitat distribution models in ecology. Ecological Modelling, 135, 147–186.

[ece32654-bib-0018] Guo, Q. , & Liu, Y. (2010). ModEco: An integrated software package for ecological niche modeling. Ecography, 33(4), 637–642.

[ece32654-bib-0019] Hanley, J. A. , & McNeil, B. J. (1982). The meaning and use of the area under a receiver operating characteristic (ROC) curve. Radiology, 143(1), 29–36. 7063747 10.1148/radiology.143.1.7063747

[ece32654-bib-0020] Hanski, I. (1991). Single‐species metapopulation dynamics: Concepts, models and observations. Biological Journal of the Linnean Society, 42, 17–38.

[ece32654-bib-0021] Hanspach, J. , Kühn, I. , Pompe, S. , & Klotz, S. (2010). Predictive performance of plant species distribution models depends on species traits. Perspectives in Plant Ecology, Evolution and Systematics, 12, 219–225.

[ece32654-bib-0022] Hanspach, J. , Schweiger, O. , Kühn, I. , Plattner, M. , Pearman, P. B. , Zimmermann, N. E. , & Settele, J. (2014). Host plant availability potentially limits butterfly distributions under cold environmental conditions. Ecography, 37(3), 301–308.

[ece32654-bib-0023] Hernandez, P. A. , Graham, C. H. , Master, L. L. , & Albert, D. L. (2006). The effect of sample size and species characteristics on performance of different species distribution modeling methods. Ecography, 29, 773–785.

[ece32654-bib-0024] Hirzel, A. H. , & Le Lay, G. (2008). Habitat suitability modelling and niche theory. Journal of Applied Ecology, 45, 1372–1381.

[ece32654-bib-0025] Husband, B. C. , & Barrett, S. C. H. (1998). Spatial and temporal variation in population size of *Eichhornia paniculata* in ephemeral habitats: Implications for metapopulation dynamics. Journal of Ecology, 86(6), 1021–1031.

[ece32654-bib-0026] Jones, C. C. , Acker, S. A. , & Halpern, C. B. (2010). Combining local‐and large‐scale models to predict the distributions of invasive plant species. Ecological Applications, 20, 311–326. 20405790 10.1890/08-2261.1

[ece32654-bib-0027] Jost, L. (2007). Partitioning diversity into independent alpha and beta components. Ecology, 88(10), 2427–2439. 18027744 10.1890/06-1736.1

[ece32654-bib-0028] Kunin, W. E. , & Gaston, K. J. (1993). The biology of rarity: Patterns, causes and consequences. Trends in Ecology and Evolution, 8(8), 298–301. 21236173 10.1016/0169-5347(93)90259-R

[ece32654-bib-0029] Lawson, C. R. , Hodgson, J. A. , Wilson, R. J. , & Richards, S. A. (2014). Prevalence, thresholds and the performance of presence–absence models. Methods in Ecology and Evolution, 5, 54–64.

[ece32654-bib-0030] Liu, C. , Berry, P. M. , Dawson, T. P. , & Pearson, R. G. (2005). Selecting thresholds of occurrence in the prediction of species distributions. Ecography, 28, 385–393.

[ece32654-bib-0031] Liu, C. , White, M. , & Newell, G. (2013). Selecting thresholds for the prediction of species occurrence with presence‐only data. Journal of Biogeography, 40(4), 778–789.

[ece32654-bib-0060] Liu, C. , Berry, P. M. , Dawson, T. P. , & Pearson, R. G. (2005). Selecting thresholds of occurrence in the prediction of species distributions. Ecography, 28(3), 385–393.

[ece32654-bib-0061] Liu, C. , Newell, G. , & White, M. (2016). On the selection of thresholds for predicting species occurrence with presence‐only data. Ecology and Evolution, 6(1), 337–348. doi: 10.1002/ece3.1878 26811797 10.1002/ece3.1878 PMC4716501

[ece32654-bib-0032] Lobo, J. M. , Jiménez‐Valverde, A. , & Real, R. (2008). AUC: A misleading measure of the performance of predictive distribution models. Global Ecology and Biogeography, 17(2), 145–151.

[ece32654-bib-0033] Lomba, A. , Pellissier, L. , Randin, C. , Vicente, J. , Moreira, F. , Honrado, J. , & Guisan, A. (2010). Overcoming the rare species modelling paradox: A novel hierarchical framework applied to an Iberian endemic plant. Biological Conservation, 143(11), 2647–2657.

[ece32654-bib-0034] Manel, S. , Williams, H. C. , & Ormerod, S. J. (2001). Evaluating presence–absence models in ecology: The need to account for prevalence. Journal of Applied Ecology, 38, 921–931.

[ece32654-bib-0035] McPherson, J. M. , Jetz, W. , & Rogers, D. J. (2004). The effects of species' range sizes on the accuracy of distribution models: Ecological phenomenon or statistical artefact? Journal of Applied Ecology, 41, 811–823.

[ece32654-bib-0036] Murphy, A. H. , & Daan, H. (1985). Forecast evaluation In Murphy A. H., & Katz R. W. (Eds.), Probability, statistics, and decision making in the atmospheric sciences (pp. 379–437). Boulder: Westview Press.

[ece32654-bib-0037] Papeş, M. , & Gaubert, P. (2007). Modelling ecological niches from low numbers of occurrences: Assessment of the conservation status of poorly known viverrids (Mammalia, Carnivora) across two continents. Diversity and Distributions, 13(6), 890–902.

[ece32654-bib-0038] Pásztor, L. , Botta‐Dukát, Z. , Magyar, G. , Meszena, G. , & Czárán, T. (2016). Theory‐based ecology: A Darwinian approach. Oxford: Oxford University Press.

[ece32654-bib-0064] Pearce, J. , & Ferrier, S. (2000). Evaluating the predictive performance of habitat models developed using logistic regression. Ecological Modelling, 133(3), 225–245.

[ece32654-bib-0039] Peirce, C. S. (1884). The numerical measure of the success of predictions. Science, 4(93), 453–454. 10.1126/science.ns-4.93.453-a 17795531

[ece32654-bib-0040] Pliscoff, P. , Luebert, F. , Hilger, H. H. , & Guisan, A. (2014). Effects of alternative sets of climatic predictors on species distribution models and associated estimates of extinction risk: A test with plants in an arid environment. Ecological Modelling, 288, 166–177.

[ece32654-bib-0041] Pontius, R. G. Jr , & Millones, M. (2011). Death to Kappa: Birth of quantity disagreement and allocation disagreement for accuracy assessment. International Journal of Remote Sensing, 32, 4407–4429.

[ece32654-bib-0042] Powers, D. M. (2011). Evaluation: From precision, recall and F‐measure to ROC, informedness, markedness and correlation. Journal of Machine Learning Technologies, 2, 37–63.

[ece32654-bib-0043] R Core Team (2014). R: A language and environment for statistical computing. R Foundation for Statistical Computing, Vienna, Austria. Retrieved from http://www.R-project.org/.

[ece32654-bib-0044] Randin, C. F. , Dirnböck, T. , Dullinger, S. , Zimmermann, N. E. , Zappa, M. , & Guisan, A. (2006). Are niche‐based species distribution models transferable in space? Journal of Biogeography, 33, 1689–1703.

[ece32654-bib-0045] Ribeiro, D. , Somodi, I. , & Čarni, A. (2016). Transferability of a predictive *Robinia pseudacacia* distribution model in northeast Slovenia. Geografski Zbornik/Acta Geographica Slovenica, 56(1), 25–43.

[ece32654-bib-0046] Robertson, M. P. , Peter, C. I. , Villet, M. H. , & Ripley, B. S. (2003). Comparing models for predicting species' potential distributions: A case study using correlative and mechanistic predictive modelling techniques. Ecological Modelling, 164(2), 153–167.

[ece32654-bib-0047] Santika, T. (2011). Assessing the effect of prevalence on the predictive performance of species distribution models using simulated data. Global Ecology and Biogeography, 20, 181–192.

[ece32654-bib-0048] Shabani, F. , Kumar, L. , & Ahmadi, M. (2016). A comparison of absolute performance of different correlative and mechanistic species distribution models in an independent area. Ecology and Evolution, 6(16), 5973–5986. 27547370 10.1002/ece3.2332 PMC4983607

[ece32654-bib-0049] Thuiller, W. , Lafourcade, B. , Engler, R. , & Araújo, M. B. (2009). BIOMOD—A platform for ensemble forecasting of species distributions. Ecography, 32, 369–373.

[ece32654-bib-0050] Tilman, D. , May, R. M. , Lehman, C. L. , & Nowak, M. A. (1994). Habitat destruction and the extinction debt. Nature, 371(6492), 65–66.

[ece32654-bib-0051] Wilks, D. S. (2011). Statistical methods in the atmospheric sciences, Vol. 100 New York: Academic press.

[ece32654-bib-0052] Williams, J. N. , Seo, C. , Thorne, J. , Nelson, J. K. , Erwin, S. , O'Brien, J. M. , & Schwartz, M. W. (2009). Using species distribution models to predict new occurrences for rare plants. Diversity and Distributions, 15, 565–576.

[ece32654-bib-0053] Wisz, M. S. , Hijmans, R. J. , Li, J. , Peterson, A. T. , Graham, C. H. , Guisan, A. & NCEAS Predicting Species Distributions Working Group (2008). Effects of sample size on the performance of species distribution models. Diversity and Distributions, 14(5), 763–773.

[ece32654-bib-0054] Youden, W. J. (1950). Index for rating diagnostic tests. Cancer, 3(1), 32–35. 15405679 10.1002/1097-0142(1950)3:1<32::aid-cncr2820030106>3.0.co;2-3

[ece32654-bib-0055] Zimmermann, N. E. , Edwards, T. C. , Moisen, G. G. , Frescino, T. S. , & Blackard, J. A. (2007). Remote sensing‐based predictors improve distribution models of rare, early successional and broadleaf tree species in Utah. Journal of Applied Ecology, 44, 1057–1067. 18642470 10.1111/j.1365-2664.2007.01348.x PMC2368764

[ece32654-bib-0056] Zimmermann, N. E. , Yoccoz, N. G. , Edwards, T. C. , Meier, E. S. , Thuiller, W. , Guisan, A. , … Pearman, P. B. (2009). Climatic extremes improve predictions of spatial patterns of tree species. Proceedings of the National Academy of Sciences, 106(Supplement 2), 19723–19728. 10.1073/pnas.0901643106 PMC2780931 19897732

[ece32654-bib-0057] Zurell, D. , Grimm, V. , Rossmanith, E. , Zbinden, N. , Zimmermann, N. E. , & Schröder, B. (2012). Uncertainty in predictions of range dynamics: Black grouse climbing the Swiss Alps. Ecography, 35, 590–603.

